# Omeprazole improves chemosensitivity of gastric cancer cells by m^6^A demethylase FTO-mediated activation of mTORC1 and DDIT3 up-regulation

**DOI:** 10.1042/BSR20200842

**Published:** 2021-01-27

**Authors:** Shuitu Feng, Guoqin Qiu, Lihong Yang, Lihua Feng, Xin Fan, Fang Ren, Kaida Huang, Yide Chen

**Affiliations:** 1 Department of Oncology, Xiamen Haicang Hospital, No. 89 Haiyu Road, Xiamen 361026, Fujian Province, People’s Republic of China; 2 Department of Oncology, Chenggong Hospital Affiliated to Xiamen University, China; 3 Department of internal medicine, Xiamen Navy Hospital, China

**Keywords:** autophagy, DDIT3, FTO, gastric cancer, m6A modification, Omeprazole

## Abstract

The curative effect for patients with advanced gastric cancer is still unsatisfactory. Proton pump inhibitors could be a promising treatment strategy that could sensitize gastric cancer cells to antitumor drugs further; however, the underlying molecular mechanism remains to be further elucidated. In this research, it was found that omeprazole pretreatment could enhance the inhibitory effect of 5-Fu, DDP and TAX on gastric cancer cells. Interestingly, omeprazole pretreatment enhanced the total m^6^A level of cells due to the decreased FTO. TCGA analysis showed that FTO expression is up-regulated in GC tissues and is negatively correlated with disease-free survival of GC patients. It was also found that FTO inhibition induced by omeprazole enhanced the activation of mTORC1 signal pathway that inhibited the prosurvival autophagy so as to improve the antitumor efficiency of chemotherapeutic drugs on GC cells. Meanwhile, transcript level of DDIT3, which is an apoptosis-related tumor suppressor gene downstream of mTORC1, was regulated by omeprazole-induced FTO silence through an m^6^A-dependent mechanism. The present study, for the first time, found that m^6^A modification and its eraser FTO may play a role in the improvement of chemosensitivity mediated by proton pump inhibitor omeprazole.

## Introduction

Gastric cancer (GC) is one of the most common cancers all over the word that is currently the second-leading cause of cancer related death among both men and women in China [1]. In recent years, significant improvements including surgery, radiation therapy, chemotherapy, targeted therapy or immunotherapy alone or in combination have been made in the treatment measures for gastric cancer [2]. However, the curative effect for patients with advanced gastric cancer is still unsatisfactory. Hence, a better understanding of the factors and potential molecular mechanism involved in improving the response efficiency of GC cells to therapeutic methods has very important clinical significance.

Proton pump inhibitors (PPIs), such as omeprazole, esomeprazole and pantoprazole (PPZ), have been widely used for the treatment of acid-related diseases, such as gastro-esophageal reflux disease, peptic ulcer disease, and the eradication of *Helicobacter pylori* infection [3]. Previously, the conclusion that PPIs could function as a promising treatment strategy for gastric cancer due to their significantly sensitize gastric cancer cells to antitumor drugs has been made by our research group and others [4–6]. It was found that PPIs directly inhibited cancer cell proliferation, drug resistance-induced metastasis and regulate stemness of GC cancer cells [5,7,8].

N6-methyladenosine (m^6^A) is reported to be the most common modification among RNA modifications. In recent years, with the improvement of experimental techniques, m^6^A has been found to be involved in the control of various cell functions. M^6^A exerts its regulatory function mainly through three kinds of proteins: RNA methylates (writers) and demethylates (erasers) and a third group of ´readers’ bind to m6A sites and determine the fate of the modified mRNA [9]. However, up to now, little has been reported on whether m^6^A modification participated in the antineoplastic drugs sensitivity of cancer cells.

Fat mass and obesity associated (FTO) is one of the first identified m^6^A eraser proteins that has an important role in promoting the occurrence of GC, and it may be a vital molecular marker in the diagnosis and prognosis of GC patients. Down-regulation of FTO significantly inhibited the proliferation, migration and invasion of GC cell lines [10,11].

In this research, for the first time, we found that omeprazole pretreatment could enhance the inhibitory effect of 5-Fu, DDP and TAX on gastric cancer cells. Omeprazole pretreatment promoted the total m^6^A level of gastric cancer cells due to the FTO inhibition induced by omeprazole. FTO inhibition induced by omeprazole enhanced the activation of mTORC1 signal pathway that inhibited the prosurvival autophagy so as to improve the antitumor efficiency of chemotherapeutic drugs on GC cells. Meanwhile, omeprazole induced FTO inhibition could increase the transcript level of DDIT3, which is an apoptosis-related tumor suppressor gene downstream of mTORC1 signaling through an m^6^A-dependent mechanism.

## Materials and methods

### Cell lines and cell culture

The human GC cell lines, AGS and HGC27, were purchased from the Hunan Yearth Biotechnology Co., Ltd. (Changsha, China). Both cell lines were cultured in RPMI-1640 medium supplemented with 10% fetal bovine serum (FBS; Gibco, Canada) and antibiotics (1% penicillin/ and100 μg/ml streptomycin sulfates, Selleck, China) in a humidified atmosphere of 95% air and 5% CO_2_ at 37°C. Proton pump inhibitor omeprazole, antitumor drugs 5-Fu, DDP, TAX and autophagy pharmacological agents 3-MA and Rapamycin were all obtained from Selleckchem (Shanghai, China).

### MTT assay

Cells were pretreated with 40 μg/ul omeprazole overnight, then, about 5000 cells were seeded in 96-well plates with 100 μl of medium with1μM 5-Fu, 10 mg/l DDP, 100 nM TAX for another 24 h. The relative cell survival rate was assessed using MTT assay according to manufacturer’s instructions.

### Cell apoptosis analysis with flow cytometer

Annexin V-FITC/propidium iodide (PI) staining assay was used to detect apoptosis of GC cells according to manufacturer’s instructions (Kengen biotech, China). Finally, apoptosis cells were assessed by gating PI and Annexin V-positive cells on a fluorescence-activated cell-sorting (FACS) flow cytometer (BD Pharmingen U.S.A.).

### Analysis of *in vitro* drug interaction

The coefficient of drug interaction (CDI) was calculated according to previous studies [12,13], and CDI value <1, =1 or >1 indicates that the drugs are synergistic, additive or antagonistic, respectively.

### GFP-RFP-LC3 puncta assessment

Gastric cancer cells that have undergone various treatments after infected with AAV- mRFP-GFP-LC3 (Hanbio, Wuhan, China) were fixed with 4% paraformaldehyde and then mounted onto microscope slides with FluorSave™ Reagent (Calbiochem, U.S.A.). Localization of RFP-LC3 or GFP-LC3 was evaluated and observed using a fluorescent inverted microscope (motic).

### Gene silencing and overexpression in GC cells

In order to construct FTO loss-of-function cell models, three siRNA sequences targeting FTO were designed and synthesized in genepharma biotech (Suzhou, China), and the one with best interference efficiency in GC cells was used for subsequent experiments. The sequence information of FTO-siRNA was: 5′-GCACAAGCATGGCTGCTTA-3′. To obtain FTO gain-of-function cell models, FTO overexpressed lentivirus particles was purchased from genepharma biotech (Suzhou, China). Relative transfection and infection experiment was performed according to manufacturer’s instructions.

### Measurement of total m^6^A level

Total RNA from cell lines of different treatment were extracted using TRIzol reagent (Invitrogen, CA) and treated with deoxyribonuclease I (Sigma, Shanghai, China). After RNA quality inspection, the commercial m^6^A RNA methylation quantification kit (ab185912; Abcam, U.S.A.) was used to detect the total m^6^A level. Briefly, 250 ng RNAs were seeded in each well, followed by adding capture antibody solution and detection antibody solution according to the manufacturer’s instruction. The m^6^A levels were then measured colorimetrically by reading the absorbance of each well at a wavelength of 450 nm.

### RNA isolation and qRT-PCR

Total RNAs were extracted from different group of cells using the TRIzol Plus RNA Purification Kit (Invitrogen, Carlbad, CA, U.S.A.). Quantitative real-time PCR was performed to detect the relative mRNA levels of genes of interest using QPCR ThermoScript One-Step System (Invitrogen, Carlbad, CA, U.S.A.) in the LC480 Real-Time PCR Detection System (Roche) according to the manufacturer’s instructions. All tests were carried out with three independent experiments. The primer sequences are listed in Table 1. The quantification analysis for relative mRNA levels of target genes was performed using the relative quantification comparative CT method.

**Table 1:** Inhibitory effects of omeprazole and antitumor drugs on the GC cells

Drug	Concentration	Growth inhibitory effect	CDI
HGC-27			
5-Fu	1μM	0.631 ± 0.008	
Ome+5-Fu	40 μg/μl + 1 μM	0.691 ± 0.011	0.956 ± 0.030
TAX	100 nM	0.260 ± 0.020	
Ome+TAX	40 μg/μl + 100 nM	0.498 ± 0.032	0.776 ± 0.029
DDP	10 mg/l	0.524 ± 0.012	
Ome+DDP	40 μg/μl + 10 mg/l	0.599 ± 0.003	0.963 ± 0.037
AGS			
5-Fu	1 μM	0.383 ± 0.074	
Ome+5-Fu	40 μg/μl + 1 μM	0.633 ± 0.008	0.653 ± 0.069
TAX	100 nM	0.229 ± 0.037	
Ome+TAX	40 μg/μl + 100 nM	0.591 ± 0.007	0.579 ± 0.030
DDP	10 mg/l	0.253 ± 0.012	
Ome+DDP	40 μg/μl + 10 mg/l	0.593 ± 0.019	0.593 ± 0.015

Drug interaction was measured as described in materials and methods.

CDI<1 indicates a synergistic effect, CDI = 1 indicates an additive effect, CDI>1 indicates an antagonistic effect (Table 1).

### Western blotting

Western blot was performed to detect the target protein expression of interest according to the methods described in the literature [14]. The primary antibodies we used were anti-p62 (18420-1-AP, Proteintech, China), anti-LC3 (18725-1-AP, Proteintech, China), anti-Bax (50599-2-lg, Proteintech, China), anti-caspase-3 (12789-1-AP, Proteintech, China), anti-S6 (#2217, CST, U.S.A.), anti-phospho-S6 (Ser235/236) (#4856, CST, U.S.A.), 4EBP1 (#9452, CST, U.S.A.), phospho-4EBP1(T37/46) (#2855, CST, U.S.A.).

### Gene-specific m^6^A qPCR

Real-time quantitative PCR (qPCR) was performed to assess the relative abundance of the selected mRNA in m^6^A antibody IP samples and input samples between the Lv-FTO, siRNA-FTO cell lines and NC cell lines. The gene-specific m^6^A qPCR was performed to determine the m^6^A abundance on the transcripts according to the method and steps reported in the previous literature [15].

### Statistical analysis

All experiments were repeated for three times and experiments were performed in triplicate. Statistical analyses were performed using GraphPad Prism 7.00 (GraphPad Software, La Jolla, CA, U.S.A.). Student’s *t* tests were used for comparison between the treatment and control groups. One-way ANOVA with Tukey’s post test for multiple comparisons was used when comparing more than two groups. For all analyses, a *P* value of ≤ 0.05 was considered significant.

## Results

### Omeprazole pretreatment inhibited chemotherapy-induced autophagy and improve the chemosensitivity of GC cells

**Figure 1:**
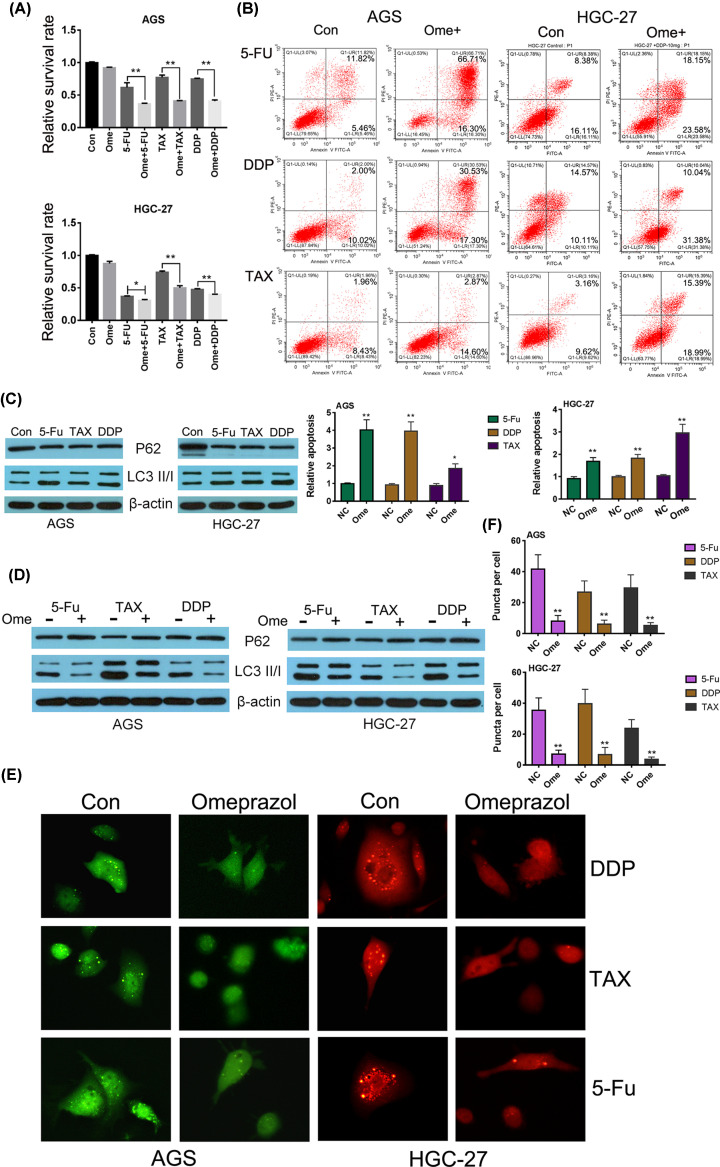
Omeprazole pretreatment inhibited chemotherapy-induced autophagy and improve the chemosensitivity of GC cells (**A**) MTT assay was performed to evaluate the relative survival rate of AGS and HGC-27 cells treated with three kinds of different antitumor drugs with or without omeprazole preconditioning. *P<0.05 versus cells in corresponding groups; **P<0.01 versus cells in corresponding groups (Con: cells without any special treatments). (**B**) Annexin V-FITC/propidium iodide (PI) staining assay was used to detect apoptosis of GC cells. (**C**) Western blot assay was used to estimate the protein expression levels of autophagy-related markers in antitumor drug-treated GC cells (Con: cells without any special treatments). (**D**) Western blot assay was used to estimate the protein expression levels of autophagy-related markers in antitumor drug-treated GC cells with or without omeprazole preconditioning. (**E**) LC3-GFP (in AGS) or LC3-RFP (in HGC-27) puncta was used to analyze the autophagy condition of lung cancer cells treated as indicated. The representative images of GC cells treated with antitumor drugs with or without omeprazole were shown. The magnification was 400×; meanwhile, the number of puncta per cell was counted (**F**); **P<0.01 versus corresponding NC groups.

Gastric adenocarcinoma cell lines AGS and HGC-27 were treated with 5-Fu, DDP and TAX for 24 h with or without omeprazole pretreatment. The results showed that three antitumor drugs can significantly reduce the relative survival rate of AGS and HGC-27 cells. At the same time, the relative survival and apoptosis rate of cells pretreated with omeprazole was significantly lower and higher respectively than that of cells untreated with omeprazole. This result suggested that, to some extent, omeprazole preconditioning could improve the response of gastric cancer cells to antitumor drugs (Table 1; Figure 1A,B). In recent years, more and more literatures have confirmed that antitumor drugs induced prosurvival autophagy of tumor cells that is a key reason for reducing chemosensitivity. Our research also confirmed the conclusion using Western blot detection of autophagic substrate P62 and classic autophagic marker LC3. Antitumor drugs reduced the expression of P62 and increased the ratio of LC3II/I (Figure 1C).

In order to explore whether omeprazole was associated with autophagy regulation in the antitumor synergism of gastric cancer cells, Western blot and LC3 puncta detections were performed in cells pretreated with or without omeprazole before antitumor drug treatment. When cells were pretreated with omeprazole, the expression of autophagic substrate P62 was relatively higher, while the ratio of LC3II/I was relatively lower compared with cells without omeprazole pretreatment (Figure 1D). Besides, in cells with omeprazole pretreatment, the number of LC3 puncta reduced obviously, and the fluorescence intensity was also weaker when compared with cells without omeprazole pretreatment (Figure 1E,F).

### N6-methyladenosine demethylase FTO is a potential target of omeprazole in GC cells

**Figure 2:**
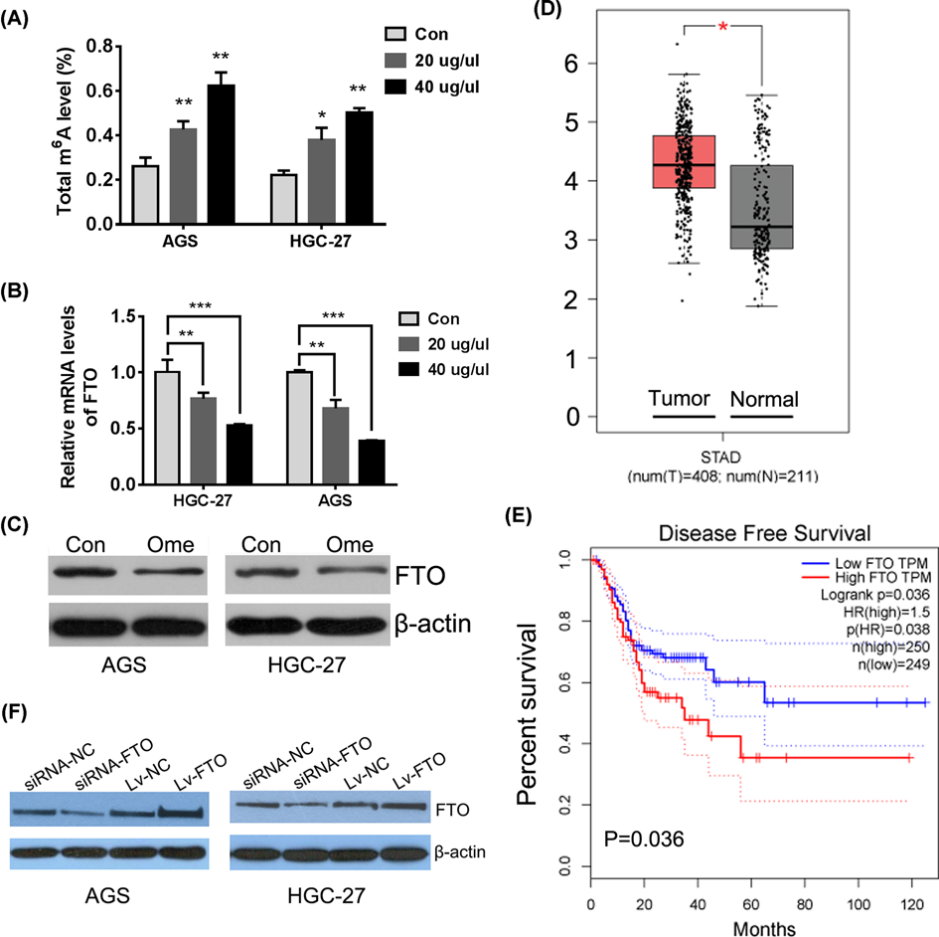
N6-methyladenosine demethylase FTO is a potential target of omeprazole in GC cells (**A**) The total mRNA m^6^A levels in GC cell samples with or without omeprazole treatment were determined by colorimetric method, *P<0.05; **P<0.01,***P<0.001 versus Con (Con: cells without any special treatments). (**B**) Relative mRNA level of FTO was confirmed by qRT-PCR in GC cell samples with or without omeprazole treatment, **P<0.01 versus cells in corresponding groups (Con: cells without any special treatments). (**C**) Expression of FTO in GC cells with or without omeprazole treatment were confirmed by Western blot. (**D**) FTO was up-regulated in GC tissues (TCGA data, Red box for tumor tissue, *n*=408; gray box for normal tissue, *n*=211). (**E**) FTO expression was negatively correlated with disease-free survival in GC patients (TCGA data, high expression, red curve, *n*=250; low expression, blue curve, *n*=249). (**F**) knockdown and overexpression of FTO were validated in both GC cells using Western blot.

m^6^A is one of RNA modifications which is involved in the control of various cell functions, such as tumor progression [16,17] and stem cell development [18]. However, little research has been done on whether m^6^A modification is involved in the regulation of chemosensitivity. Based on this idea, we tried to find out whether omeprazole preconditioning could change the total m^6^A level of gastric cancer cells first. What is amazing is that omeprazole treatment caused a significant concentration-dependent increase in the total m^6^A level of both AGS and HGC-27 cells (Figure 2A).

Then, the relative mRNA levels of classic m^6^A regulators were detected using qRT-PCR. The results showed that omeprazole treatment reduced the expression of m^6^A eraser FTO significantly, which was consistent with the promotion of total m^6^A level in both AGS and HGC-27 cells (Figure 2B). Western blot was performed to further confirm the expression of FTO, and a consistent conclusion was reached (Figure 2C). FTO expression was reported to have important roles in promoting gastric cancer occurrence and could be an vital molecular marker in the diagnosis and prognosis of GC patients [11]. We evaluated the expression and significance of FTO in overall survival (OS) and disease-free survival (DFS) in GC through GEPIA database (http://gepia.cancer-pku.cn). It was found that FTO expression is significantly elevated in GC tissues (T) compared with noncancerous tissues (N). Meanwhile, stronger FTO protein expression was associated with lower disease-free survival rate in GC patients (Figure 2D,E). In order to further analyze the function of FTO, the knockdown and overexpression models of FTO were constructed and validated in both GC cells (Figure 2F).

### FTO could restore the autophagy inhibition induced by omeprazole pretreatment and reduce the sensitivity of GC cells to antineoplastic drugs

**Figure 3:**
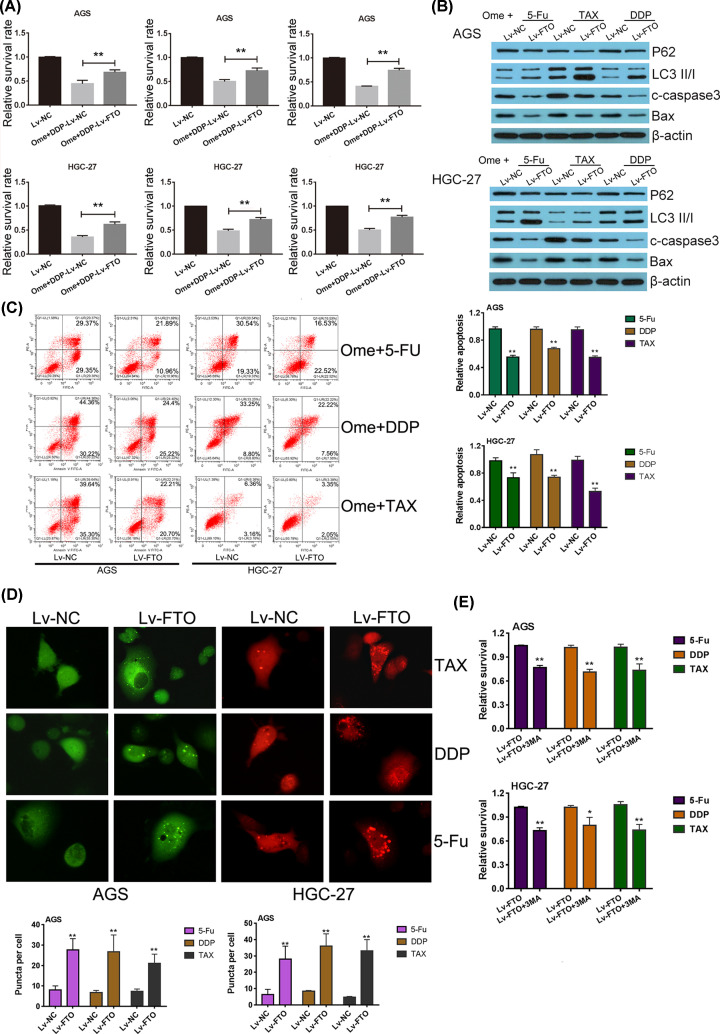
FTO could restore the autophagy inhibition induced by omeprazole pretreatment and reduce the sensitivity of GC cells to antineoplastic drugs (**A**) MTT assay was performed to evaluate the relative survival rate of FTO gain-of-function cell models treated with three kinds of different antitumor drugs with or without omeprazole preconditioning. **P<0.01 versus cells in indicated groups (Con: cells without any special treatments). (**B**) Western blot assay was used to estimate the protein expression levels of autophagy-related and apoptosis markers in FTO gain-of-function cell models treated with antitumor drugs. (**C**) Then cell apoptosis was detected using flow cytometry with annexin V FITC/ PI double staining in FTO gain-of-function cell models treated with three kinds of different antitumor drugs with omeprazole preconditioning. Meanwhile, the relative apoptosis was statistically analyzed, **P<0.01 versus NC cells of corresponding group. (**D**) FTO enhancement resulted in LC3 puncta further accumulation in AGS and HGC-27 cells treated with antitumor drugs. The representative images of LC3 puncta in GC cells were shown, the magnification was 400× and the number of puncta per cell was counted, **P<0.01 versus NC cells in corresponding groups. (**E**) MTT assay was performed to evaluate the relative survival rate of FTO gain-of-function cell models treated with 3-MA along with omeprazole preconditioning; **P<0.01 versus Lv-NC cells.

To further confirm that FTO is one of the targets of omeprazole on GC cells, gain-of-function cell models were established in both AGS and HGC-27 cells. It was found that, when FTO was overexpressed, the enhanced chemosensitivity of GC cells induced by omeprazole pretreatment significantly reduced and the relative survival rate of cells increased significantly accompanied by decreased cell apoptosis (Figure 3A,B). Because the present study has clarified that omeprazole pretreatment inhibited chemotherapy-induced prosurvival autophagy and improve the chemosensitivity of GC cells. So does FTO, the target of omeprazole, have the ability to regulate autophagy and apoptosis? First, Western blot was performed to detect the classic autophagy marker proteins in FTO gain-of-function cell models treated with antitumor drugs with omeprazole preconditioning. The results showed that when FTO was enhanced, autophagic substrate P62 decreased and the ratio of LC3II/I up-regulated obviously compared with negative control cells treated with antitumor drugs with omeprazole preconditioning (Figure 3C). Meanwhile, the number of LC3 puncta in FTO overexpressed GC cells increased obviously when compared with negative control cells without FTO up-regulation (Figure 3D). Second, Western blot was also performed to detect the classic apoptosis-related proteins in FTO gain-of-function cell models treated with antitumor drugs with omeprazole preconditioning. The results showed that when FTO was upregulated, apoptotic executive protein cleaved caspase-3 and proapoptosis protein Bax were both decreased obviously (Figure 3C). When we tried to block the autophagy of FTO-overexpressed cells again with autophagy inhibitor 3-MA, the synergistic inhibitory effect of omeprazole and antineoplastic drugs appeared again (Figure 3E). The above results suggested that FTO could restore the prosurvival autophagy inhibition induced by omeprazole pretreatment and reduce the sensitivity of GC cells to antineoplastic drugs.

### FTO/mTORC1 signal axis played roles in omeprazole induced autophagy mediation

**Figure 4:**
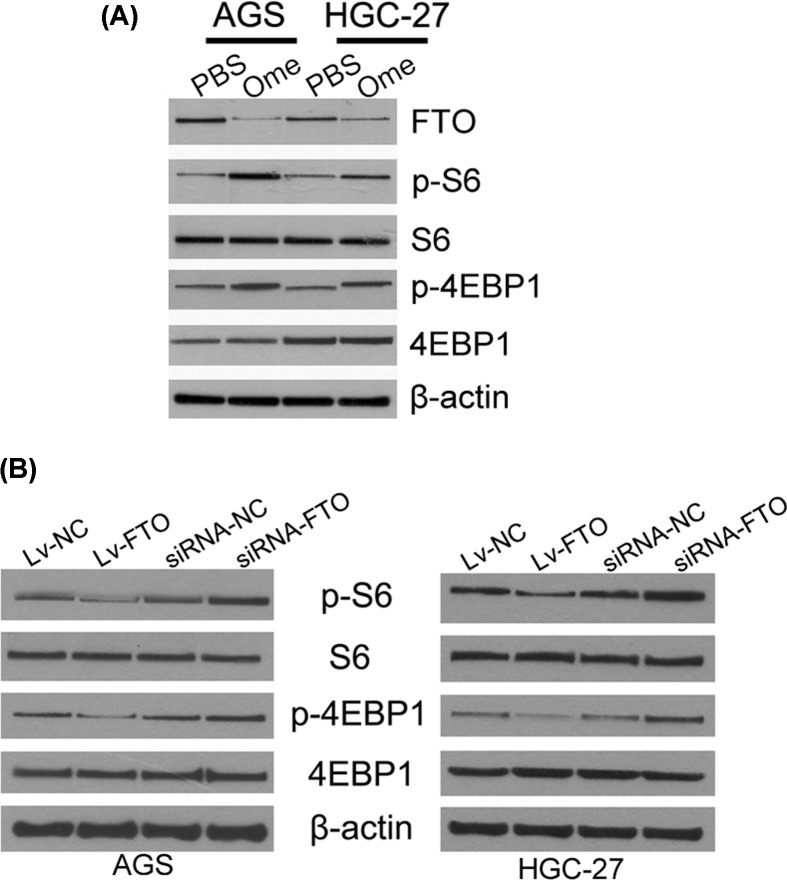
FTO / mTORC1 signal axis played roles in omeprazole induced autophagy mediation (**A**) Omeprazole treatment enhanced the p-S6 (Ser235/236) and p-4EBP1 (Ser37/46) when compared with PBS-treated cells in both AGS and HGC-27 cells. (**B**) FTO knockdown resulted in the p-S6 (Ser235/236) and p-4EBP1 (Ser37/46) promoted. In the other hand, p-S6 (Ser235/236) and p-4EBP1 (Ser37/46) attenuate when FTO was overexpressed when compared with PBS-treated cells in AGS and HGC-27 cells different cell models.

mTORC1 plays a role in switching the prosurvival autophagy [19]. Our results found that omeprazole treatment could enhance the phosphorylation S6 (Ser235/236) and 4EBP1 (Ser37/46), both of which are downstream targets of mTORC1 [20], suggested that omeprazole treatment activated mTORC1 to some extent (Figure 4A). Besides, when FTO was silenced, the phosphorylation S6 (Ser235/236) and 4EBP1 (Ser37/46) promoted. In the other hand, the phosphorylation S6 (Ser235/236) and 4EBP1 (Ser37/46) attenuate when FTO was overexpressed (Figure 4B). These results demonstrated that FTO/mTORC1 signal axis played roles in omeprazole induced autophagy mediation.

### Omeprazole/FTO/m^6^A axis regulates DDIT3 expression to promote cell apoptosis

**Figure 5:**
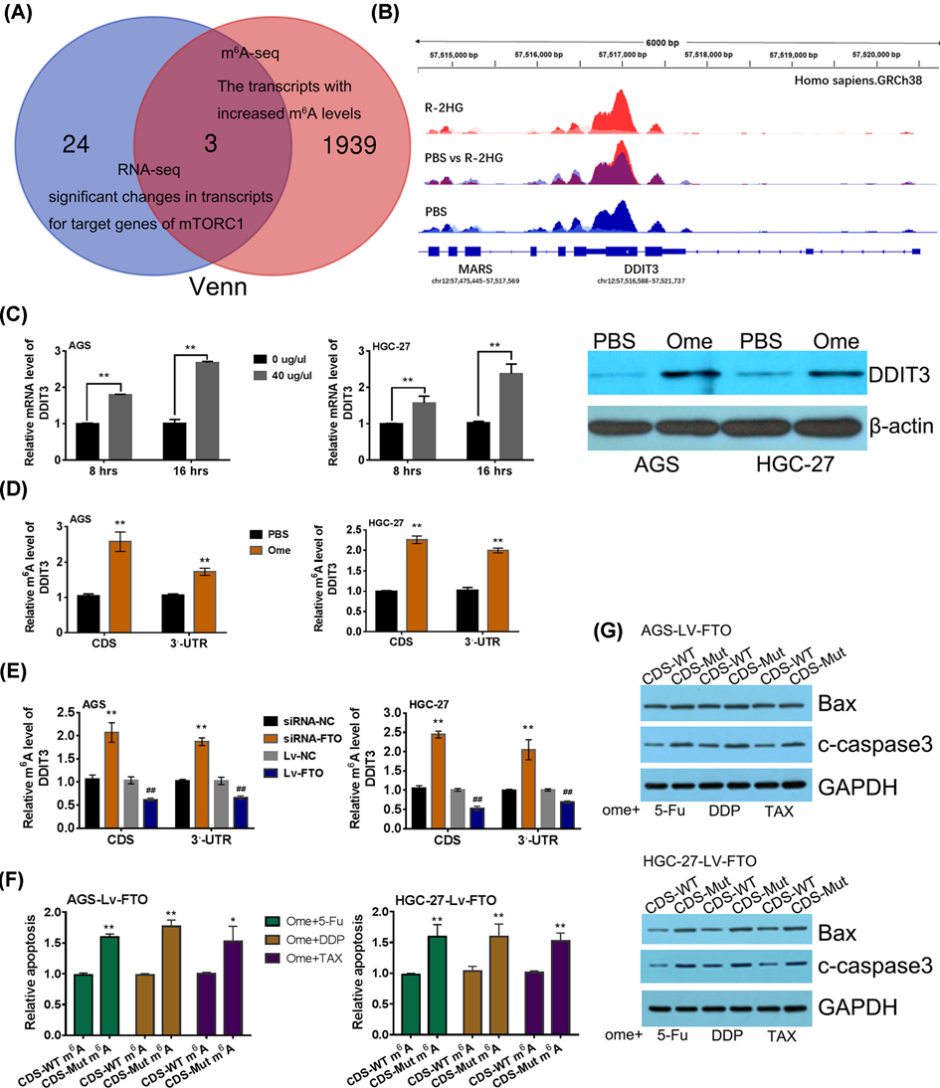
Omeprazole/FTO/m^6^A axis regulates DDIT3 expression to promote cell apoptosis (**A**) Venn diagram of significant changed transcripts of mTORC1 signal pathway genes that were concluded by bioinformatics analysis from GSEA Data Sets GSEA 87190 (m6A-seq), GSEA 87187 and GSEA 87189 (RNA-seq). (**B**) The m^6^A peaks (*P*<0.05) upon R-2HG treatment (red) versus PBS-treatment (blue) of DDIT3. (**C**) The relative mRNA levels of DDIT3 upon omeprazole treatment (40 μg) versus PBS-treatment (0 μg). **P<0.01 versus PBS-treated cells in corresponding groups. (**D**) Relative m^6^A levels of DDIT3 mRNA were evaluated using m6A-specific qPCR upon omeprazole treatment versus PBS-treatment GC cells. **P<0.01 versus PBS-treated cells in corresponding groups. (**E**) Gene-specific m6 A qPCR validation of m^6^A level changes of DDIT3 mRNA in FTO gain or loss-of-functional GC cell models. **P<0.01 versus siRNA-NC cells and *^##^P*<0.01 versus Lv-NC cells. (**F**) Relative apoptosis of FTO-overexpressed cells with or without CDS-mut m^6^A sites. *P<0.05 and **P<0.01 versus with wild-type (WT) m^6^A sites in CDS region. (**G**) The expression of apoptosis related proteins was evaluated using Western blot in both FTO overexpressed GC cells.

It was reported that when FTO was inhibited by R-2HG treatment, the transcripts with increased m^6^A levels (m^6^A-hyper) were also significantly enriched for target genes of mTORC1 signaling pathway [21]. This aroused our strong interest. Through the comparison and analysis of GSE data sets (GSEA 87190, GSEA 87187 and GSEA 87189), 3 mTORC1 pathway target genes were identified that may be regulated by FTO in m^6^A-dependent manner (Figure 5A). Besides, based on m^6^A-seq data GSEA87190, when FTO was inhibited by R-2HG, the m^6^A abundance of DDIT3 mRNA increased, especially in the CDS and 3′-UTR regions (Figure 5B). Subsequently, qRT-PCR and Western blot were performed in omeprzole pretreated GC cells. It was confirmed in AGS and HGC-27 cells. DDIT3, an apoptosis-related tumor suppressor gene downstream of mTORC1 signaling, showed significant enhancement when compared with cells without ome-treatment (Figure 5C). To explore whether ome-induced DDIT3 up-regulation is mediated through m^6^A-dependent manner in AGS and HGC-27 cells studied in this research, gene-specific m^6^A qPCR assays were performed to evaluate relative m^6^A level of DDIT3 in omeprazole pretreated GC cells. The results showed that omeprazole treatment significantly promoted the relative m^6^A level of DDIT3 in both 3′UTR and CDS regions in GC cells compared with PBS-treated cells. However, the m^6^A level of DDIT3 did not increase significantly when FTO overexpressed GC cells (Lv-FTO) were pretreated with omeprazole (Figure 5D). In order to further certificate that FTO negatively regulates DDIT3 through m^6^A-dependent manner, relative m^6^A levels of DDIT3 in different FTO functional GC cells were assessed. The results demonstrated that FTO silence could enhance m^6^A levels of DDIT3 in both CDS and 3′UTR region. Conversely, FTO overexpression obviously attenuated m^6^A levels of DDIT3 in both CDS and 3′UTR region (Figure 5E).

DDIT3 was an apoptosis-related tumor suppressor gene downstream of mTORC1 signaling. Omeprazole/FTO/m^6^A modification axis up-regulated DDIT3 expression. Thus, is this related to the synergistic effect of omeprazole and antineoplastic drugs to GC cells? To clarify the problem, the apoptosis of FTO overexpressed GC cells harboring DDIT3 CDS containing wild-type or mutant m^6^A sites (m^6^A was replaced with T) was measured. The results showed that when GC cells were treated with omeprazole in combination with antineoplastic drugs, the attenuated cell apoptosis resulted from FTO up-regulation was significantly restored due to the putative m^6^A sites were mutated in DDIT3 CDS region (Figure 5F). Correspondingly, the expression of apoptotic marker proteins c-caspase 3 and Bax was also enforced when compared with wild type group cells (Figure 5G).

## Discussion

Several researches demonstrated that PPIs was promising antitumor and chemosensitizing efficacy that could restore chemosensitivity in drug-resistant cancer cells due to tumor acidity was considered as key determinant of drug-resistance and tumor progression [22]. A few studies suggested that PPI combined with chemotherapeutic drugs can synergistically inhibit the production of endometrial cancer and melanoma [23,24]. To our knowledge, whether drug combination has synergistic effect should be judged by the coefficient of drug interaction (CDI) value. CDI value <1, =1 or >1 indicates that the drugs are synergistic, additive or antagonistic, respectively. Based on this, in the present study, it was reconfirmed that omeprazole preconditioning could indeed produce synergistic inhibitory effect on gastric cancer cells with antitumor drugs on the basis of CDI value.

The potential functional role of m^6^A-related genes in gastric cancer (GC) prognosis has not been systematically researched. At the same time, little research has been done on whether m^6^A modification is involved in the regulation of chemosensitivity. FTO was reported to enhance the chemoresistance of cervical squamous cell by reducing m^6^A levels of β-catenin mRNA [25]. Based on this, we hypothesize whether the increased chemosensitivity of gastric cancer cells induced by omeprazole preconditioning is related to m^6^A modification.

The results of colorimetric assay showed that omeprazole treatment could significantly promote the overall m^6^A level of GC cells which implied that omeprazole pretreatment leads to the desregulation of some m^6^A related genes. So, we detected mRNA levels of a series of m^6^A classical related factors by qRT-PCR. It was found that after omeprazole treatment, m^6^A eraser FTO decreased significantly, which was in line with the result that the overall proportion of m^6^A increased after omeprazole treatment. However, when our team attempted to reconfirm the result that the overall level of m^6^A alteration from the blood samples collected clinically, the low quality of RNA from blood sources led to the failure of the validation.

Up to now, little research has been done on the involvement of m^6^A-related factors in autophagy regulation. However, it is worth mentioning that research has identified that m^6^A eraser FTO as an oncogenic role via autophagy. In addition, FTO was considered or associated with autophagy regulation [26,27]. Our results suggested that FTO promoted survival-promoting autophagy of gastric cancer cells, at least in the context of the present study.

R-2HG was a FTO inhibitor, after R-2HG treatment, the transcripts with increased m^6^A levels (m^6^A-hyper) were also significantly enriched for target genes in mTORC1 signaling pathways [21]. The mTORC1 signal that was reported to coordinate the autophagy and apoptosis was first thought of by us [19]. This result demonstrated that FTO could negatively regulate mTORC1 activation that could in turn inhibit autophagy. Further, based on the bioinformatics analysis of data, we identified three mTORC1 pathway target genes that may be regulated by FTO in m^6^A-dependent manner. Among them, DDIT3 which is an apoptosis-related tumor suppressor gene was found to be up-regulated with the down-regulation of FTO. Based on these, it was speculated omeprazole induced FTO inhibition could increase the transcript level of DDIT3, which is an apoptosis-related tumor suppressor gene downstream of mTORC1 signaling through an m^6^A-dependent mechanism. Fortunately, we have also substantiated this scientific hypothesis through experiments.

In our study, the molecular mechanism of the effect of omeprazole on GC cells through regulating FTO / mTORC1 signal axis includes two aspects: first, FTO inhibition induced by omeprazole enhanced the activation of mTORC1 signal pathway that inhibited the prosurvival autophagy so as to improve the cell apoptosis on GC cells induced by chemotherapeutic drugs. In this part, we basically confirmed the omeprazole mainly to inhibit prosurvival autophagy to promote apoptosis by setting autophagy inhibitor 3-MA group (we only measured the survival capacity to laterally reflect the cell apoptosis, but did not directly detect the cell apoptosis, this was a deficiency).

Second, It was found that omeprazole-induced FTO silence could activate mTORC1, then up-regulated DDIT3 through an m6A-dependent mechanism. DDIT3 is an apoptosis-related tumor suppressor gene which directly promote the apoptosis of tumor cells. However, whether DDIT3 could promote the apoptosis of GC cells by regulating autophagy deserves further study.

## Data Availability

The datasets used and/or analyzed during the current study are available from the corresponding author on reasonable request.

## Abbreviations

CDI: coefficient of drug interaction
DFS: disease-free survival
GC: gastric cancer
OS: overall survival
PPI: proton pump inhibitor
PPZ: pantoprazole

## References

[1] Zhang B., Ling T., Zhaxi P., Cao Y., Qian L., Zhao D. et al. (2019) Proton pump inhibitor pantoprazole inhibits gastric cancer metastasis via suppression of telomerase reverse transcriptase gene expression. Cancer Lett. 452, 23–30 10.1016/j.canlet.2019.03.029 30910586

[2] Bai T.L., Liu Y.B. and Li B.H. (2019) MiR-411 inhibits gastric cancer proliferation and migration through targeting SETD6. Eur. Rev. Med. Pharmacol. Sci. 23, 3344–3350 31081088 10.26355/eurrev_201904_17697

[3] Freedberg D.E., Kim L.S. and Yang Y.X. (2017) The Risks and Benefits of Long-term Use of Proton Pump Inhibitors: Expert Review and Best Practice Advice From the American Gastroenterological Association. Gastroenterology 152, 706–715 10.1053/j.gastro.2017.01.031 28257716

[4] Chen M., Lu J., Wei W., Lv Y., Zhang X., Yao Y. et al. (2018) Effects of proton pump inhibitors on reversing multidrug resistance via downregulating V-ATPases/PI3K/Akt/mTOR/HIF-1alpha signaling pathway through TSC1/2 complex and Rheb in human gastric adenocarcinoma cells in vitro and in vivo. OncoTargets Therapy 11, 6705–6722 10.2147/OTT.S161198 30349304 PMC6188003

[5] Feng S., Zheng Z., Feng L., Yang L., Chen Z., Lin Y. et al. (2016) Proton pump inhibitor pantoprazole inhibits the proliferation, selfrenewal and chemoresistance of gastric cancer stem cells via the EMT/betacatenin pathways. Oncol. Rep. 36, 3207–3214 10.3892/or.2016.5154 27748935

[6] Huang S., Chen M., Ding X., Zhang X. and Zou X. (2013) Proton pump inhibitor selectively suppresses proliferation and restores the chemosensitivity of gastric cancer cells by inhibiting STAT3 signaling pathway. Int. Immunopharmacol. 17, 585–592 10.1016/j.intimp.2013.07.021 23973653

[7] Guan X.W., Zhao F., Wang J.Y., Wang H.Y., Ge S.H., Wang X. et al. (2017) Tumor microenvironment interruption: a novel anti-cancer mechanism of Proton-pump inhibitor in gastric cancer by suppressing the release of microRNA-carrying exosomes. Am. J. Cancer Res. 7, 1913–1925 28979813 PMC5622225

[8] Zhang B., Yang Y., Shi X., Liao W., Chen M., Cheng A.S. et al. (2015) Proton pump inhibitor pantoprazole abrogates adriamycin-resistant gastric cancer cell invasiveness via suppression of Akt/GSK-beta/beta-catenin signaling and epithelial-mesenchymal transition. Cancer Lett. 356, 704–712 10.1016/j.canlet.2014.10.016 25449432

[9] Guo M., Liu X., Zheng X., Huang Y. and Chen X. (2017) m(6)A RNA Modification Determines Cell Fate by Regulating mRNA Degradation. Cell. Reprog. 19, 225–231 10.1089/cell.2016.0041 28682669

[10] Li Y., Zheng D., Wang F., Xu Y., Yu H. and Zhang H. (2019) Expression of Demethylase Genes, FTO and ALKBH1, Is Associated with Prognosis of Gastric Cancer. Dig. Dis. Sci. 64, 1503–1513 10.1007/s10620-018-5452-2 30637548 PMC6522448

[11] Xu D., Shao W., Jiang Y., Wang X., Liu Y. and Liu X. (2017) FTO expression is associated with the occurrence of gastric cancer and prognosis. Oncol. Rep. 38, 2285–2292 10.3892/or.2017.5904 28849183

[12] Chen L., Ye H.L., Zhang G., Yao W.M., Chen X.Z., Zhang F.C. et al. (2014) Autophagy inhibition contributes to the synergistic interaction between EGCG and doxorubicin to kill the hepatoma Hep3B cells. PLoS ONE 9, e85771 10.1371/journal.pone.0085771 24465696 PMC3897495

[13] Wang W., Chen D. and Zhu K. (2018) SOX2OT variant 7 contributes to the synergistic interaction between EGCG and Doxorubicin to kill osteosarcoma via autophagy and stemness inhibition. J. Exp. Clin. Cancer Res.: CR 37, 37 10.1186/s13046-018-0689-3 29475441 PMC6389193

[14] Zhu L., Huang F., Wan T., Xu H. and Zhao Q. (2018) Overexpression of long noncoding RNA LINC00882 is associated with poor prognosis in hepatocellular carcinoma. OncoTargets Therapy 11, 5209–5217 10.2147/OTT.S170825 30271163 PMC6145351

[15] Liu Y., Wang R., Zhang L., Li J., Lou K. and Shi B. (2017) The lipid metabolism gene FTO influences breast cancer cell energy metabolism via the PI3K/AKT signaling pathway. Oncol. Lett. 13, 4685–4690 10.3892/ol.2017.6038 28599470 PMC5452952

[16] Chen M., Wei L., Law C.T., Tsang F.H., Shen J., Cheng C.L. et al. (2018) RNA N6-methyladenosine methyltransferase-like 3 promotes liver cancer progression through YTHDF2-dependent posttranscriptional silencing of SOCS2. Hepatology 67, 2254–2270 10.1002/hep.29683 29171881

[17] Dai D., Wang H., Zhu L., Jin H. and Wang X. (2018) N6-methyladenosine links RNA metabolism to cancer progression. Cell Death Dis. 9, 124 10.1038/s41419-017-0129-x 29374143 PMC5833385

[18] Nishizawa Y., Konno M., Asai A., Koseki J., Kawamoto K., Miyoshi N. et al. (2018) Oncogene c-Myc promotes epitranscriptome m(6)A reader YTHDF1 expression in colorectal cancer. Oncotarget 9, 7476–7486 10.18632/oncotarget.23554 29484125 PMC5800917

[19] Yang H., Wen Y., Zhang M., Liu Q., Zhang H., Zhang J. et al. (2019) MTORC1 coordinates the autophagy and apoptosis signaling in articular chondrocytes in osteoarthritic temporomandibular joint. Autophagy 1–18 31007149 10.1080/15548627.2019.1606647 PMC6984599

[20] Villar V.H., Nguyen T.L., Delcroix V., Teres S., Bouchecareilh M., Salin B. et al. (2017) mTORC1 inhibition in cancer cells protects from glutaminolysis-mediated apoptosis during nutrient limitation. Nat. Commun. 8, 14124 10.1038/ncomms14124 28112156 PMC5264013

[21] Su R., Dong L., Li C., Nachtergaele S., Wunderlich M., Qing Y. et al. (2018) R-2HG Exhibits Anti-tumor Activity by Targeting FTO/m(6)A/MYC/CEBPA Signaling. Cell 172, 90e23–105e23 10.1016/j.cell.2017.11.031 29249359 PMC5766423

[22] Cao Y., Chen M., Tang D., Yan H., Ding X., Zhou F. et al. (2018) The proton pump inhibitor pantoprazole disrupts protein degradation systems and sensitizes cancer cells to death under various stresses. Cell Death Dis. 9, 604 10.1038/s41419-018-0642-6 29789637 PMC5964200

[23] Azzarito T., Venturi G., Cesolini A. and Fais S. (2015) Lansoprazole induces sensitivity to suboptimal doses of paclitaxel in human melanoma. Cancer Lett. 356, 697–703 10.1016/j.canlet.2014.10.017 25449440

[24] Song T., Jeon H.K., Hong J.E., Choi J.J., Kim T.J., Choi C.H. et al. (2017) Proton Pump Inhibition Enhances the Cytotoxicity of Paclitaxel in Cervical Cancer. Cancer Res. Treatment: Off. J. Korean Cancer Assoc. 49, 595–606 10.4143/crt.2016.034 PMC5512380 27669706

[25] Zhou S., Bai Z.L., Xia D., Zhao Z.J., Zhao R., Wang Y.Y. et al. (2018) FTO regulates the chemo-radiotherapy resistance of cervical squamous cell carcinoma (CSCC) by targeting beta-catenin through mRNA demethylation. Mol. Carcinog. 57, 590–597 10.1002/mc.22782 29315835

[26] Chen J., Wang C., Fei W., Fang X. and Hu X. (2019) Epitranscriptomic m6A modification in the stem cell field and its effects on cell death and survival. Am. J Cancer Res. 9, 752–764 31106001 PMC6511641

[27] Jin S., Zhang X., Miao Y., Liang P., Zhu K., She Y. et al. (2018) m(6)A RNA modification controls autophagy through upregulating ULK1 protein abundance. Cell Res. 28, 955–957 10.1038/s41422-018-0069-8 30046135 PMC6123428

